# Characteristics of BeiDou Navigation Satellite System Multipath and Its Mitigation Method Based on Kalman Filter and Rauch-Tung-Striebel Smoother

**DOI:** 10.3390/s18010198

**Published:** 2018-01-12

**Authors:** Qiuzhao Zhang, Wei Yang, Shubi Zhang, Xin Liu

**Affiliations:** School of Environment Science and Spatial Informatics, China University of Mining and Technology, NO. 1 Daxue Road, Xuzhou 221000, China; qiuzhaocumt@163.com (Q.Z.); ts16160034a3tm@cumt.edu.cn (W.Y.); cumtlx2015@163.com (X.L.)

**Keywords:** multipath, BDS, Kalman filters and RTSS, EMD, wavelet, single difference residuals

## Abstract

Global Navigation Satellite System (GNSS) carrier phase measurement for short baseline meets the requirements of deformation monitoring of large structures. However, the carrier phase multipath effect is the main error source with double difference (DD) processing. There are lots of methods to deal with the multipath errors of Global Position System (GPS) carrier phase data. The BeiDou navigation satellite System (BDS) multipath mitigation is still a research hotspot because the unique constellation design of BDS makes it different to mitigate multipath effects compared to GPS. Multipath error periodically repeats for its strong correlation to geometry of satellites, reflective surface and antenna which is also repetitive. We analyzed the characteristics of orbital periods of BDS satellites which are consistent with multipath repeat periods of corresponding satellites. The results show that the orbital periods and multipath periods for BDS geostationary earth orbit (GEO) and inclined geosynchronous orbit (IGSO) satellites are about one day but the periods of MEO satellites are about seven days. The Kalman filter (KF) and Rauch-Tung-Striebel Smoother (RTSS) was introduced to extract the multipath models from single difference (SD) residuals with traditional sidereal filter (SF). Wavelet filter and Empirical mode decomposition (EMD) were also used to mitigate multipath effects. The experimental results show that the three filters methods all have obvious effect on improvement of baseline accuracy and the performance of KT-RTSS method is slightly better than that of wavelet filter and EMD filter. The baseline vector accuracy on east, north and up (E, N, U) components with KF-RTSS method were improved by 62.8%, 63.6%, 62.5% on day of year 280 and 57.3%, 53.4%, 55.9% on day of year 281, respectively.

## 1. Introduction

Recently, the application of Global Navigation Satellite System (GNSS) technology in deformation monitoring has become an important way to monitor the structural health of buildings for its advantages in automation, all-weather, real time and large scale, etc. [1]. Affected by orbit error, ionospheric delay, tropospheric delay, phase center offset and multipath error, the accuracy of GNSS carrier phase measurement generally only reaches centimeter level. To achieve the millimeter or even submillimeter accuracy which is necessary for deformation monitoring, strict elimination of errors is essential. In the case of short baseline (<3 km), most errors excluding multipath can be eliminated to a large extent by the carrier phase double difference (DD) technique because of the high space correlation.

During the last two decades, many GPS multipath mitigation methods have been developed which can be classified to three classes [2]. The first one is selecting an open area with no reflection or shelter for GNSS measurement while it is limited by objective conditions in deformation monitoring. The second one is improving the hardware devices of GNSS receivers to exclude multipath errors when the GNSS signal is used. The last and most common method is data post-processing approaches, including weight adjustment based on data signal to noise ratio (SNR) techniques [3]; ray-tracing approach [4]; and sidereal filtering (SF) based on the coordinate domain [5,6]/observation domain [7,8] method, which is the most popular.

At present, the BeiDou Navigation Satellite System (BDS) consisting of geostationary earth orbit (GEO) satellites, inclined geosynchronous orbit (IGSO) satellites and medium Earth orbit (MEO) satellites can provide high-accuracy location services, which are comparable to Global Position System (GPS) in the Asian-Pacific region [9,10]. Many literatures have researched the performance of the combination of BDS/GPS [11,12]. By 2020, the service range of BDS will cover all the world and its data processing has become a hot spot and focus of GNSS positioning. It is necessary to conduct accuracy assessments and to develop error cancellation techniques about BDS positioning such as multipath mitigation.

Since the relative geometry of a GPS satellite with receiver and reflective surface repeats itself in each sidereal day, multipath errors are highly correlated between consecutive sidereal days if the geometry changes are the same [13,14]. Hence, the SF based on coordinate domain using daily repetition of multipath can extract the multipath model from the coordinate sequence of the first day to correct that of the next day at a fixed station. However, this method uses the average satellite orbital period and ignores the differences between satellites, so it cannot be effectively applied in BDS multipath mitigation with its three different types of orbital satellites. Axelrad et al. found that the orbital repeat periods of GPS satellites are not strictly a sidereal day (about 23 h and 56 min) and there is a time shift which is not a constant for different satellites [15]. Therefore, the SF based on observation domain, which calculates the orbital period of each satellite, extracts and corrects the multipath model on corresponding satellite observations between the observation sequences of two multipath periods, was proposed and developed. Zhong et al. developed a SF based on single difference (SD) residuals for mitigating GPS multipath effects on short baselines [7]. Ye et al. studied the multipath repeat cycle of BDS satellites, and proposed a SF carrier phase multipath elimination approach of BDS system [14]. Dong et al. proposed a Multipath Hemispherical Map (MHM) to achieve real-time resolution and correction of multipath errors [16]. Dai et al. compared the MHM algorithm with the sidereal filtering algorithm, and proposed an improved multipath error parameterization model for BDS GEO multipath elimination [17].

In order to obtain the multipath error model, it is essential to select an effective filtering method which controls the effect of multipath elimination. Lau proposed a robust three-level wavelet packet for carrier phase multipath [18]. Zhong et al. proposed an adaptive wavelet transform based on a cross-validation method to mitigate GPS multipath effects [19]. Dai et al. applied empirical mode decomposition (EMD) in denoising coordinate sequence of short GPS baseline [20]. Kaloop et al. used four prediction models which are applied and used with neural network solutions: back-propagation, Cascade-forward back-propagation, adaptive filter and extended Kalman filter, to extract GPS time-series model and denoise the data for monitoring of structures [21]. FIR filtering has also been used in extracting multipath models in literature [22]. To obtain a more accurate multipath model, a multipath model extraction method based on Kalman filter and Rauch-Tung-Striebel Smoother (RTSS) was proposed and the multipath repetition characteristics of BDS satellites were explained by qualitative analysis and quantitative calculation in the paper. To verify the performance of the new method, we compared it with the traditional wavelet and EMD methods used in [19,20].

The paper is arranged as follows: in Section 2, the basic principle of multipath error is explained and the steps of reconstructing single difference (SD) residuals is shown with a flowchart. Qualitative analysis and quantitative calculation of orbital periods of BDS satellites are carried out in Section 3. Section 4 introduces three filter methods for denoising SD residuals for extracting multipath models and a simulation experiment was carried out. In Section 5, we verified the consistency between orbital periods and multipath periods of BDS satellites through a set of measured data, applied three filtering methods in extracting multipath models and found they all achieve good results in multipath mitigation. Finally, conclusions were drawn in Section 6.

## 2. Multipath Error Theory

### 2.1. Basic Principle

The multipath problem is a widespread problem in signal propagation, such as the network signal multipath and the sensor data delivery multipath including the underwater sonar multipath and the satellite signal multipath. Data delivery path in Information-Centric Sensor Networks was studied in [23,24]. The AUV Positioning Method Based on Tightly Coupled SINS/LBL was proposed to deal with the Underwater Acoustic Propagation Multipath in [25]. In [26], the Kalman filter was introduced to realize multi-path network synchronization. Satellite signal multipath mitigation is a key step in precise GNSS positioning and is our focus work. The path deviation of satellite signals caused by multipath effects can be expressed as [7]:

(1) $$S_{multi}=\frac{\Delta \varphi_{m}}{2\pi }\lambda =\frac{\lambda }{2\pi }\arctan(\frac{\alpha \sin(\frac{4\pi D}{\lambda }\sin\theta )}{1+\alpha \cos(\frac{4\pi D}{\lambda }\sin\theta )})$$

where *D* represents the horizontal distance between the receiver antenna and the reflector around the station, *θ* and *λ* are respectively the incidence angle and wavelength of the reflected signal, *α* is the reflection coefficient of reflector and Δ*ϕ_m_* represents the phase delay caused by multipath effects. It can be found that the multipath errors are mostly affected by horizontal distance *D*, incident angle *θ* and reflection coefficient *α*. However, the reflective surface, antenna and their properties remain unchanged in case of a static baseline, which means that the horizontal distance and reflection coefficient are constant. Hence it is only the incidence angle of reflected signals that could change multipath errors. Meanwhile, the incidence angle of reflected signals is determined by position of satellites in the sky which would periodically repeat in specific orbit, so multipath errors are also periodic. It indicates that we can mitigate multipath effects for current data by subtracting the multipath error models extracted from the last period data.

### 2.2. Integer Ambiguity Estimation and Validation with Multipath Errors

The integer ambiguity estimation and validation are the two basic steps of high precision carrier phase positioning. The least-squares ambiguity decorrelation adjustment (LAMBDA) has always been the most popular algorithm for ambiguity estimation since Teuniseen proposed it in 1990s [27]. The core idea is to reduce the search space by decorrelating the covariance matrix of float ambiguity to improve the search efficiency. Many modified algorithms were developed based on LAMBDA, such as the mixed upper/lower triangular integer Cholesky decomposition algorithm [28], inverse integer Cholesky decomposition algorithm [29] PAR LAMBDA algorithm [30]. Another representative algorithm called MLAMBDA algorithm was proposed by Chang [31] and is used for ambiguity resolution in this paper.

Another key step is ambiguity validation which can ensure the reliability of fixed ambiguity. The common methods include three types: ratio test based on statistics [32], index method of success rate/failure rate [33] and the combination of the two previous methods [34]. In this paper, the R-ratio test was applied to validate the ambiguity. The DD equations without/with multipath errors can be expressed as:

(2) y=Aa+Bb+e

(3) $$y^{\prime }=y+m=Aa+Bb+e$$

where *y*/*y*’ represent the GNSS observations without/with multipath errors, *a* and *b* represent the ambiguity vector and baseline vector with coefficient matrixes *A* and *B*, *m* and *e* are the multipath errors and DD residuals, respectively. Classifying multipath errors into DD residuals, we get:

(4) $$y=Aa+Bb+e^{\prime }$$

where *e*’ = e − *m*, then −*m* is the DD multipath errors remaining in DD residuals *e’*.

We can compute the float solutions, integer ambiguity and fixed solution using the next criteria:

(5) $$\min_{a,b}||y-Aa-Bb|{|}_{Q_{y}^{-1}}^{2}\;\mathrm{with}\;b\in R^{n}\;\mathrm{and}\;a\in R^{n}$$

(6) $$\min_{a}||\hat{a}-a|{|}_{Q_{\hat{a}}^{-1}}^{2}\;\mathrm{with}\;a\in Z^{n}$$

(7) $$\overset{⌣}{b}=\hat{b}-Q_{\hat{b}\hat{a}}Q_{\hat{a}}^{-1}(\hat{a}-\overset{⌣}{a})$$

where ${\left||M|\right|}_{N}^{2}=M^{T}NM$ and $Q_{y}^{-1}$ and $Q_{a}^{-1}$ are the variance-covariance matrixes of DD observations and DD float ambiguity, respectively. The main contribution of the LAMBDA algorithm is introducing a Z-transformation [27] by *z* = *Z^T^a* and $Q_{\hat{z}}=Z^{T}Q_{\hat{a}}Z$, then criterion (6) is changed to:

(8) $$\min_{z}||\hat{z}-z|{|}_{Q_{\hat{z}}^{-1}}^{2}\;\mathrm{with}\;z\in Z^{n}$$

Here ambiguity correlation is reduced and search efficiency is improved.

After integer ambiguity estimation the ratio test can be carried out with the following criterion [32]:

(9) $$ratio=\frac{\Omega_{\sec}}{\Omega_{\min}}$$

where Ω*_i_* $={\left||\hat{a}-{\overset{˘}{a}}_{i}|\right|}_{Q_{a}^{-1}}^{2}$ and is the quadratic form of ambiguity residuals, Ω_min_ and Ω_sec_ represent the minimum and secondary values of Ω*_i_*, respectively. The threshold is set to 3 and if ratio ≥ 3, the integer ambiguity $\overset{˘}{a}$ corresponding to Ω_min_ is considered to be fixed correctly [34].

Actually, the maximum effect on carrier phase by multipath errors is only up to 1/4 wavelengths when $\arctan(\frac{\alpha \sin(\frac{4\pi D}{\lambda }\sin\theta )}{1+\alpha \cos(\frac{4\pi D}{\lambda }\sin\theta )})$ approaches $\frac{\pi }{2}$ according to Equation (1). Two cases should be considered in ambiguity estimation: first, multipath errors are so small that they cannot change the estimation of integer ambiguity—but could affect the result of the baseline vector by changing phase observations (the non-integer part of the phase); second, multipath errors bring out a carrier phase measurement error of a few centimeters and both the ambiguity estimation and baseline vector are changed. Therefore, both two cases will be improved if multipath errors were removed from residuals.

### 2.3. Reconstruction of SD Residuals and Correction of Multipath Error

**Figure 1:**
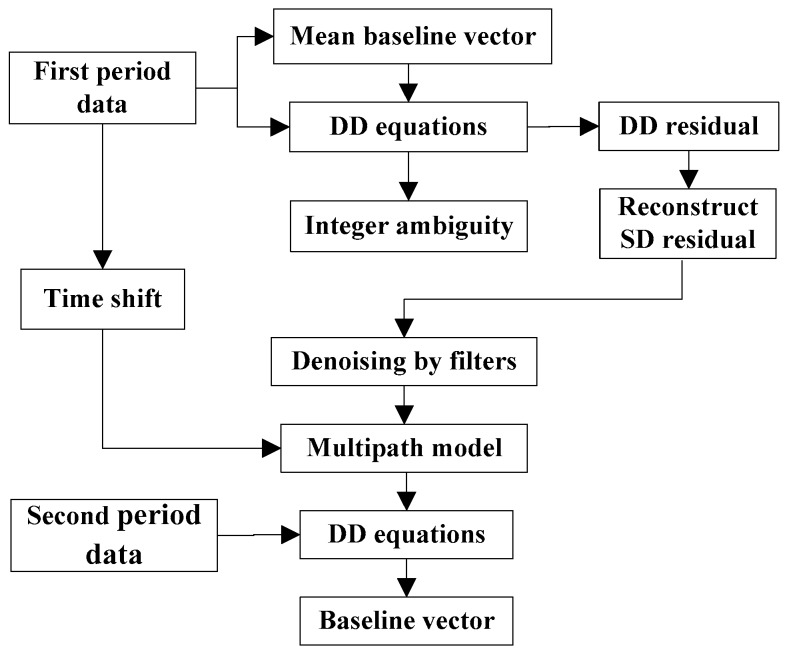
Process of reconstructing SD residuals and correcting multipath errors.

Since most model errors excluding multipath error can be eliminated by DD processing, multipath error becomes the main component of the remaining errors in DD observations. There are two disadvantages when DD residuals are applied to obtain multipath models. Firstly, the reference satellite in the DD process is the satellite with the highest elevation angle, which means that once the reference satellite changes, the DD residuals sequence will become complex and cannot correspond to the specific satellites. Secondly, it is difficult to determine the orbital period of two satellites as their orbital periods are different from each other. Reference [35] gives another way that we can reconstruct SD residuals from DD residuals by the transition matrix which contains the “zero mean” assumption $\sum^{\text{​}}w_{i}s_{AB}^{i}=0$, then the reconstructed SD residuals can be expressed as:

(10) $$\left[\begin{array}{c} s_{AB}^{1}\\ s_{AB}^{2}\\ s_{AB}^{3}\\ \ldots \\ s_{AB}^{n} \end{array}\right]=\left[\begin{array}{c} \sum w_{i}s_{AB}^{i}\\ d_{AB}^{12}\\ d_{AB}^{13}\\ \ldots \\ d_{AB}^{1n} \end{array}\right]*{\left[\begin{array}{ccccc} w_{1} & w_{2} & w_{3} & \ldots & w_{n}\\ 1 & -1 & 0 & \ldots & 0\\ 1 & & -1 & \ldots & 0\\ & & \ldots & & \\ 1 & 0 & 0 & \ldots & -1 \end{array}\right]}^{-1}$$

where $s_{AB}^{i}$ and $d_{AB}^{ij}$ represent reconstructed SD residuals and DD residuals, respectively; *w_i_* is the weighting factor. Because we use the weighting model of elevation angles, *w_i_* = sin^2^(*θ*) here. Then the process of calculating SD residuals and correcting multipath errors is as shown in Figure 1:

(1) The mean solution of the baseline vector of first period data was calculated as the true baseline vector *b*;
(2) The baseline solution is substituted back to the DD equations of first period data to compute ambiguity inversely. Then the float ambiguity solution is fixed to integer ambiguity *a*;
(3) Next, both *b* and *a* were substituted into the DD equations to obtain DD residuals consisting of multipath errors and Gaussian white noise;
(4) DD residuals were reconstructed to SD residuals by Equation (10);
(5) The multipath model of each BDS satellite was extracted from SD residuals through different filter methods with random noise being filtered;
(6) The multipath model sequences were adapted according to the time shift computed from first period data;
(7) The adaptive multipath model sequences were substituted into the SD equations of the second period data to mitigate multipath effects according to high correlation between multipath errors of two period data;
(8) Finally, the baseline coordinate sequence of the second period data was evaluated from corrected DD equations.

## 3. Orbital Periods of BDS Satellites

### 3.1. Qualitative Analysis

**Figure 2:**
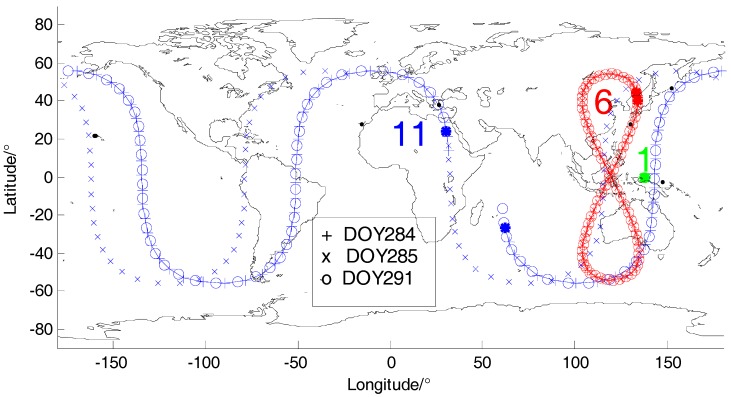
Satellite tracks of BDS GEO satellite (C01), IGSO satellite (C06) and MEO satellite (C11). Satellite traces for C06 and C11 on DOY 284, 285 and 291 are shown with the marks “+”, ”×” and “o” respectively.

**Figure 3:**
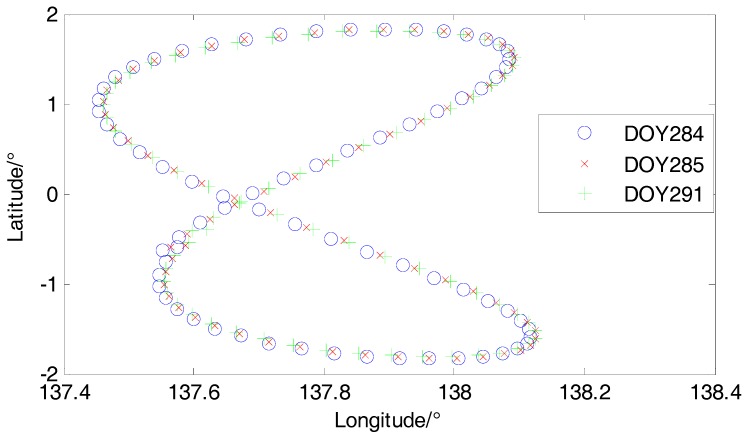
Satellite tracks of BDS GEO satellite (C01) on DOY 284, 285 and 291 with the marks “o”, ”×” and “+” respectively.

All the GPS satellites are the same orbital satellite-MEO satellite and their orbital periods are almost one sidereal day although there may be a deviation of several minutes (notice that the orbital period here is not calculated in the celestial coordinate system, but in the site-centric coordinate system) However, for the three types of satellites of BDS system, the orbital repeatability is more complex. Figure 2 shows the satellite tracks of BDS GEO satellite (C01), IGSO satellite (C06) and MEO satellite (C11) on day of year (DOY) 284, 285 and 291 in 2016. We can find that the ground tracks of the C06 is like an “8” shape and its tracks on the three days almost coincide. This indicates that the satellite orbital periods of IGSO satellites are about 1 day. Meanwhile, the tracks of C11 just coincide on DOY 284 and 291, but that on DOY 285 is independent of the other two days. Consequently, the orbital period of BDS MEO satellites is about 7 days. In Figure 2, we can also find that C01 almost remained stationary during the three days, but actually it still moved in a very small area because it was vulnerable to tidal changes, atmospheric pressure and nutation and other external intrusion. As shown in Figure 3, BDS GEO satellites will move in the shape of a small “8” within 4 degrees in latitude and 1 degree in longitude. It is clear that the orbital periods of GEO satellites are about 1 sidereal day.

### 3.2. Quantitative Calculation

After qualitative analysis of the BDS satellites orbital periods, quantitative calculation for each satellite is also needed. On one side, the orbital periods of satellites are not strictly integer days and there is a time shift. On the other hand, the orbital periods of BDS satellites are different from each other. According to the orbit radius and the angular velocity correction given by the broadcast ephemeris, the angular velocity of the satellite can be obtained at each epoch:

(11) $$n=\sqrt{GM}/a^{\frac{3}{2}}+\Delta n$$

where *GM* = 1.996498 × 10^7^ is the square root of the product of the gravitational constant of the earth and the mass of the earth, Δn is the correction parameter of satellite angular velocity, *a* is the square root of the long half axis of the satellite orbit ellipse. For BDS GEO and IGSO satellites, the time shifts can be computed by:

(12) ΔT=86,400−2π/n

We also obtain the time shifts of MEO satellites by:

(13) ΔT=7×86,400−26π/n

**Figure 4:**
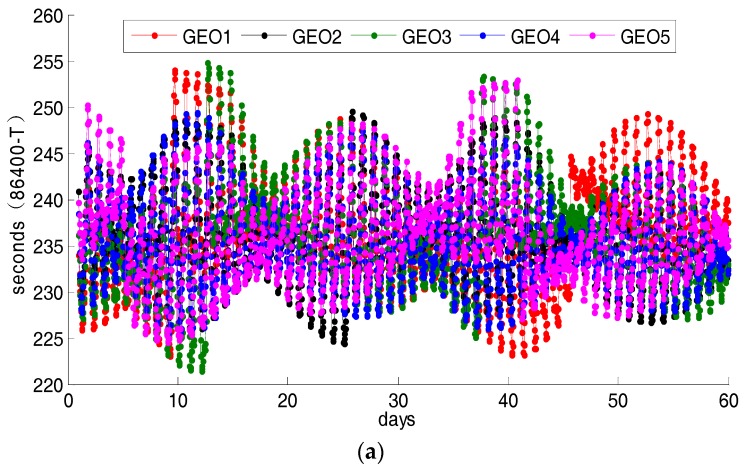

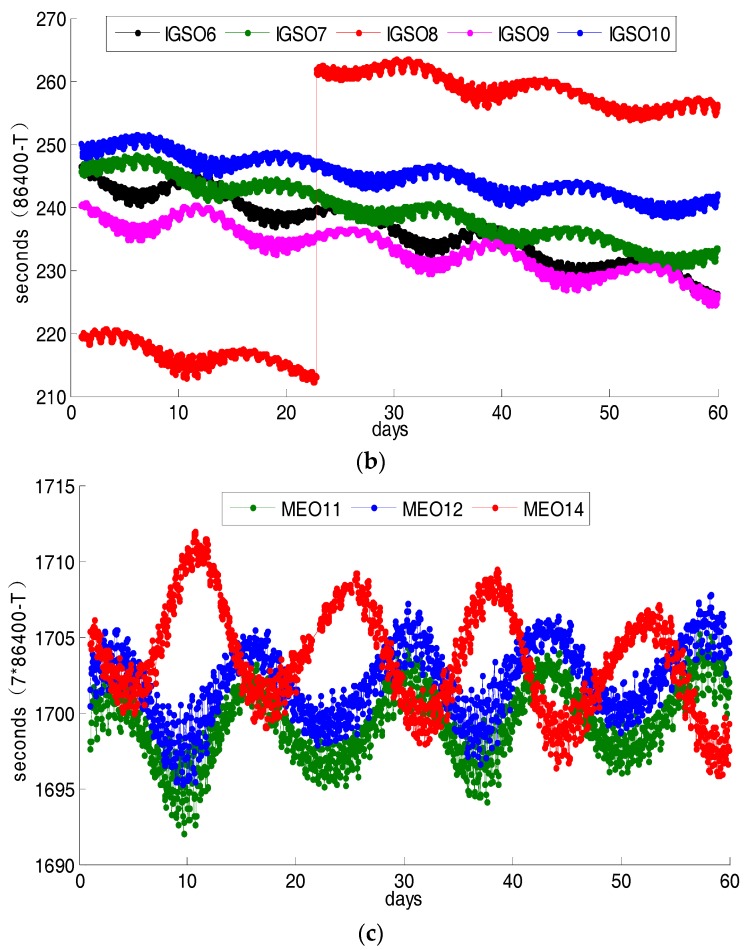
The orbital repeat periods of the BDS satellites from day 1 to 59 of the year 2017. The values in the top and middle panels are derived from 86,400 subtracting the repeat times of the GEO and IGSO satellites, respectively. The values obtained by 7 × 86,400 subtracting the repeat time of MEO are marked in the bottom panel.

Based on the above formulas and BDS ephemeris in January and February 2017, we calculated the time shifts of all satellites at each ephemeris epoch as shown in Figure 4 and Table 1. From Figure 4, it is easy to find that the time shifts of the three different types of satellites are different. The corresponding time shifts of different satellites with the same type varies at different time. Even the time shift of one satellite varies at different time. As Figure 4a shows, the time shift curves of GEO satellites fluctuate greatly even in one day and the range is about 30 s. The result also indicates that GEO satellite has a high-frequency jitter during operation due to interference from external forces. Moreover, it is easy to find that the time shifts of GEO1–GEO5 satellites come into a similar variation trend in the two months so their mean value are nearly the same values with 235 s or 236 s. In Figure 4b we can find that, the time shifts of the IGSO satellites is relatively stable in one day and there is no great volatility like GEO satellites. However, the curves of time shifts of different IGSO satellites keep independent of each other, consequently, a few seconds of deviation occurs among the time shifts of IGSO satellites. The minimum value is 233 s corresponding to IGSO9 and the maximum value is 245 s corresponding to IGSO10. Only IGSO8 happens to a great jump about 50 s on 22 January. The main reason for this phenomenon may be orbital maneuver. For the MEO satellites in Figure 4c, the change tendency of each curve fluctuates is around 1700 s although there still exist differences. However, it can be found from Table 1 that the range of time shift of MEO satellites are 12–16 s, which is smaller than that of GEO and IGSO satellites.

## 4. Filter Methods for Extracting Multipath Error Models

As mentioned in Section 2.3, the DD and SD residuals consist of multipath errors and white noise. The indispensable step of multipath mitigation is extracting multipath models from SD residuals. Denoising SD residuals to approach multipath errors is the main part of this process where reasonable filter should be selected. Many researchers have studied on filter methods for multipath mitigation such as wavelet filter, EMD filter and FIR filter. In this paper, Kalman filter and RTS smoother (KF-RTSS) was firstly used in BDS multipath mitigation. The basic principle of the wavelet filter and EMD filter were explained briefly and the process of KF-RTSS was described in detail as follows.

### 4.1. Wavelet Filter

A wavelet filter decomposes a signal into different frequency layers by Fourier transform [36]. In most cases, the low frequency layers are the useful part corresponding to the multipath errors of the SD residuals, and the high frequency layers contains white noise that is going to be filtered. Some details of the high frequency layers are still preserved by different threshold methods. Then the preserved details and low frequency part are reconstructed to approach original data. The process of wavelet filter consists of selecting wavelet basis function, determining decomposition level and defining threshold, respectively. The wavelet basis function we used is sym6, the decomposition progression is 4 and the threshold of filter is default provided by MATLAB.

### 4.2. EMD Filter

Empirical mode decomposition (EMD) is proposed by Huang in 1998 [20,37]. As a new signal decomposition method, it is a part of the Hilbert Huang transform (HHT) method which can analyze the spectrum of non-linear and non-stationary signals adaptively. EMD is mainly applied to obtain the instantaneous frequency characteristics of signal sequences. Hence it is adaptive to decompose data into a set of Intrinsic Mode Function (IMF) components with different frequencies, which represent the basis of the data. Similar to the wavelet filter, the high-frequency IMF component is considered as the noise part. Although the wavelet filter has multi-resolution and high time-frequency domain resolution, its application is limited by many problems, such as wavelet basic selection and decomposition progression determination. Reference [38] shows that the product of the energy density and the mean period of the IMF component of the Gaussian white noise signal is a constant. Hence we can determine the noise progression by Equation (14):

(14) $$R_{k}=|(ET_{k}-ET_{k-1})/(\frac{1}{k-1}\sum_{i=1}^{k-1}ET_{i})|$$

where *ET_k_* is the product of the energy density and the mean period of the IMF *k*, *R_k_* is the ratio factor that control noise progression. Generally, when *R_k_* ≥ 2, the sum of the first IMF to IMF *k* − 1 is the noise part of the original signal and the sum of other IMF components is the filtered signal. Therefore, EMD offers a different approach to data decomposition, and we can apply it to the study of extracting BDS multipath models.

### 4.3. Kalman Filter and RTS Smoother

Kalman filter is the traditional algorithm for the Kalman filter (KF) and Rauch-Tung-Strieber smoother (RTSS) can provide the minimum mean square estimates (MMSE) of states for state-space models with additive Gaussian system and observation noises given a series of the past, current, and future observations [39]. Hence, it has been widely used in data denoising processing. Wang applied KF-RTSS method in the position estimation of a GPS software receiver [40]. Sarkka et al. predicted ESTSP Competition Time Series by Unscented Kalman Filter and RTS Smoother [41]. Chiang et al. overcame the limitations of KF and improved the performance of an INS/GPS integrated system by KF-RTSS method [42]. In this paper, the KF-RTSS method is introduced to extract the BDS multipath models from SD residuals and correct multipath errors. The simplest state space model is a linear model, which can be expressed as follows:

(15) $$\mathrm{State}\;\mathrm{equation}\;x_{k}=F_{k-1}x_{k-1}+w_{k-1}$$

(16) $$\mathrm{Measurement}\;\mathrm{equation}\;z_{k}=H_{k}x_{k}+v_{k}$$

where *x_k_* is the system state of *k* step, *z_k_* is the measurement of *k* step, *F_k_*_−1_ is the transition matrix transforming the system state from the *k* − 1 step to the *K* step and *H_k_* is the transition matrix giving the relation between the observation vector *z_k_* and the state vector *x_k_*. *w_k_*_−1_ and *v_k_*_−1_ are the process noise and measurement noise of state equation and measurement equation, respectively. Meanwhile, these are theoretically white noise that meets *ε*(*w_k_*_−1_) = 0, *ε*(*v_k_*) = 0 and $\varepsilon \left(w_{k}v_{k}^{T}\right)=0$, $\varepsilon \left(w_{0}w_{k}^{T}\right)=0,$ $\varepsilon \left(x_{0}v_{k}^{T}\right)=0,$ which means measurement noise is irrelevant to system status, process noise is irrelevant to observation, and measurement noise is also irrelevant to process noise. Let $\varepsilon \left(w_{k-1}w_{k-1}^{T}\right)=Q_{k-1}$ and $\varepsilon \left(v_{k}v_{k}^{T}\right)=R_{k}$, then the iterative process of KF and RTSS is as follows:

(1) Prediction and update of state and measurement (KF)
  One-step prediction:
  (17) $${\hat{x}}_{k}=F_{k-1}x_{k-1}$$
  (18) $${\hat{z}}_{k}=H_{k}{\hat{x}}_{k}$$
  Variance matrix of one-step prediction:
  (19) $${\hat{P}}_{k}=F_{k-1}P_{k-1|}F^{T}+Q_{k-1}$$
  The state estimation is corrected by the measurement error term:
  (20) $$x_{k}={\hat{x}}_{k}+K_{k}(z_{k}-{\hat{z}}_{k})$$
  Update the variance matrix of state:
  (21) $$P_{k}={\hat{P}}_{k}-K_{k}H{\hat{P}}_{k}$$
  where *K_k_* represents the filter gain matrix of step *k*, which can be expressed as:
  (22) $$K_{k}={\hat{P}}_{k}H^{T}{\left(H{\hat{P}}_{k}H^{T}+R_{k}\right)}^{-1}$$
(2) After step *k*, the state estimator of the *K* step dynamic system is smoothed by the following formulas (RTSS):
  (23) $${\hat{x}}_{k+1}=F_{k}x_{k}$$
  (24) $${\hat{P}}_{k+1}=F_{k}P_{k}F_{k}{}^{T}+Q_{k}$$
  (25) $$C_{k}=P_{k}F_{k}{}^{T}{{\hat{P}}_{k+1}}^{-1}$$
  (26) $$x_{k}^{S}=x_{k}+C_{k}(x_{k+1}^{S}-{\hat{x}}_{k+1})$$
  (27) $$P_{k}^{S}=P_{k}+C_{k}(P_{k+1}^{S}-{\hat{P}}_{k+1})C_{k}{}^{T}$$

where, ${\hat{x}}_{k+1}$ and ${\hat{P}}_{k+1}$ are the smoother estimates for the state mean and state covariance on time step *k*. $x_{k}^{S}$ and $P_{k}^{S}$ are the filter estimates for the state mean and state covariance on time step *k*. ${\hat{x}}_{k+1}$ and ${\hat{P}}_{k+1}$ are the predicted state mean and state covariance on time step *k* + 1, which are the same as in the Kalman filter. *C_k_* is the smoother gain on time step *k*, which tells how much the smoothed estimates should be corrected on that particular time step.

**Figure 5:**
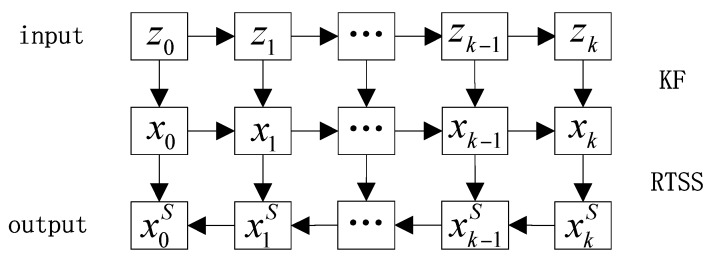
Process of KF-RTSS method.

The process of KF and RTSS is shown as Figure 5. The input SD residuals are the measurement $z_{k}$. The filtered and smoothed output of multipath models are the state $x_{k}^{S}$. The system state is filtered from step 1 to *k* and then each step is smoothed conversely according to filtered value and smoothed value of next step. The initial value of the smoothing process $x_{k}^{S}$ is equal to the last state estimation of the filtering process $x_{k}$.

### 4.4. Simulation Experiment

Because the multipath error sequence contains signals with different frequencies, we can simulate multipath signal by the combination of three sine wave signals

(28) $$S_{t}=\sin(2\pi t/200)+\sin(2\pi t/400)+\sin(2\pi t/600)$$

**Figure 6:**
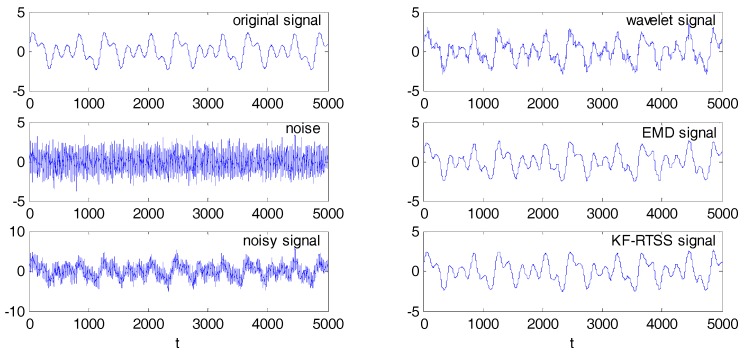
The original signal, Gauss white noise, noisy signal and filtered signal after wavelet filter, EMD filter and KF-RTSS.

A Gauss white noise with a standard deviation of 1 is added, then the data model turns to:

(29) $$M_{t}=S_{t}+e_{t}$$

where, *M_t_* is the mixed signal which can be considered as BDS SD residual sequence, *et* is the Gauss white noise which should be filtered. The sampling interval is 1 s, and the sampling number is 5000. In order to verify the denoising effect of KF-RTSS method, wavelet filter and EMD filter are also used as the comparison. As shown on the left of Figure 6, from top to bottom are the original signal, Gauss white noise and noisy signal. On the right, extracted signals by wavelet filter, EMD filter and KF-RTSS method are shown respectively. Table 2 gives the correlation coefficient between filtered signals and the original signal. The root mean square errors of both signals are assessed. It is clearly that the correlation coefficient of KF-RTSS signal and original signal is the highest (0.9927) while RMSE of EMD signal is closest to original signal. In the three filters, Wavelet signal show the worst performance. Generally, correlation coefficients between filtered signals and original signal are all more than 0.97, which indicates that three filters all have good performance on denoising the contaminated signals.

## 5. Performance Analysis of BDS Multipath Mitigation Based on the KF-RTSS Method

In order to verify the consistency between orbital period and multipath periods of BDS satellites and the feasibility of the KF-RTSS proposed, we calculated the SD residuals and extract the multipath model with respect to each satellite, and evaluate the baseline results after three filtering methods. This set of data were collected from 27 September to 10 October 2017 year with the frequency of 1 Hz and 5° elevation mask angle at the roof of School of environment and Geometrics, China University of Mining and Technology. The two GNSS receivers are Trimble R10 (Trimble Navigation, Sunnyvale, California, United States) units and one of them was set in an unobstructed environment as base station while another one was placed about 5 m away from the southeast direction of a white wall as the rover station. The length of the baseline is about 62.210 m. To ensure that the base station is not affected by multipath effects and the rover station has a clear multipath effect, we turned off the anti-multipath function of the rover station while switching on the function of the base station. The processing of extracting SD residuals and mitigating multipath errors are implemented by the GNSS data processing software developed by ourselves.

### 5.1. SD Residual of Different Types of BDS Satellites

**Figure 7:**
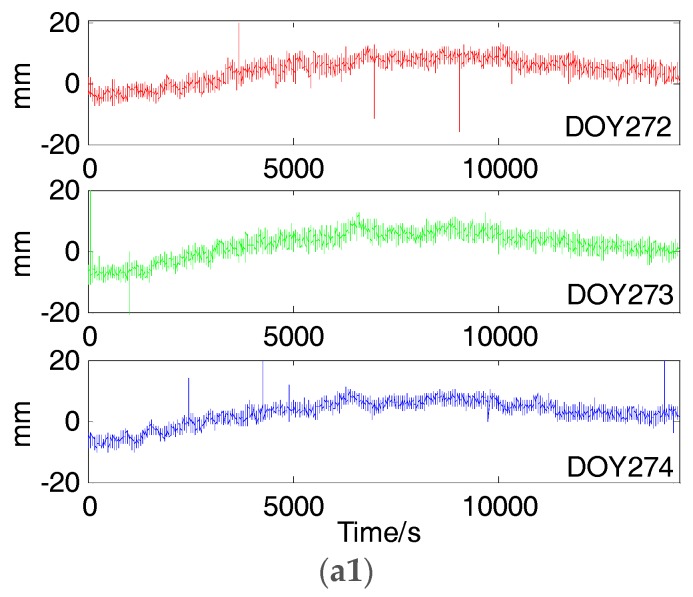

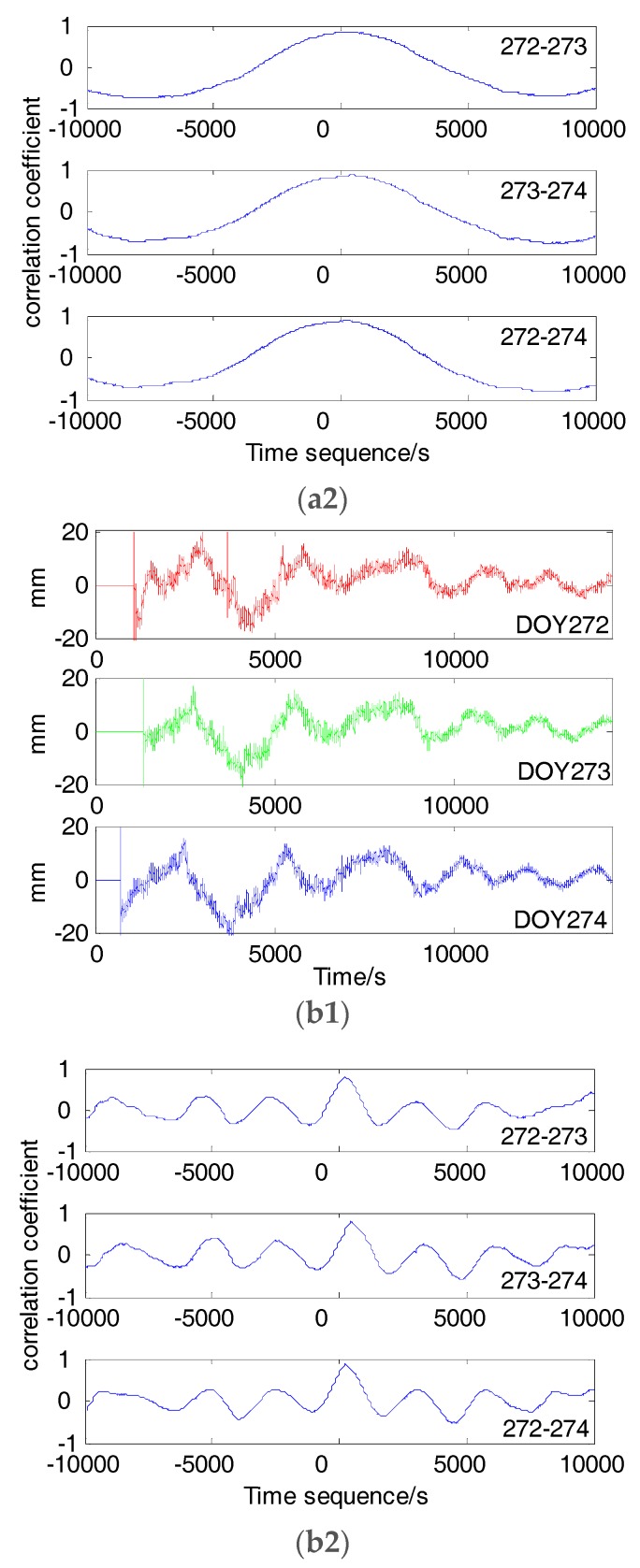
SD residuals of GEO5 (**a1**) and IGSO6 (**b1**) on DOY 272, 273 and 274 and correlation curve of GEO5 (**a2**) and GEO6 (**b2**) on 272–273, 272–274 and 273–274.

**Figure 8:**
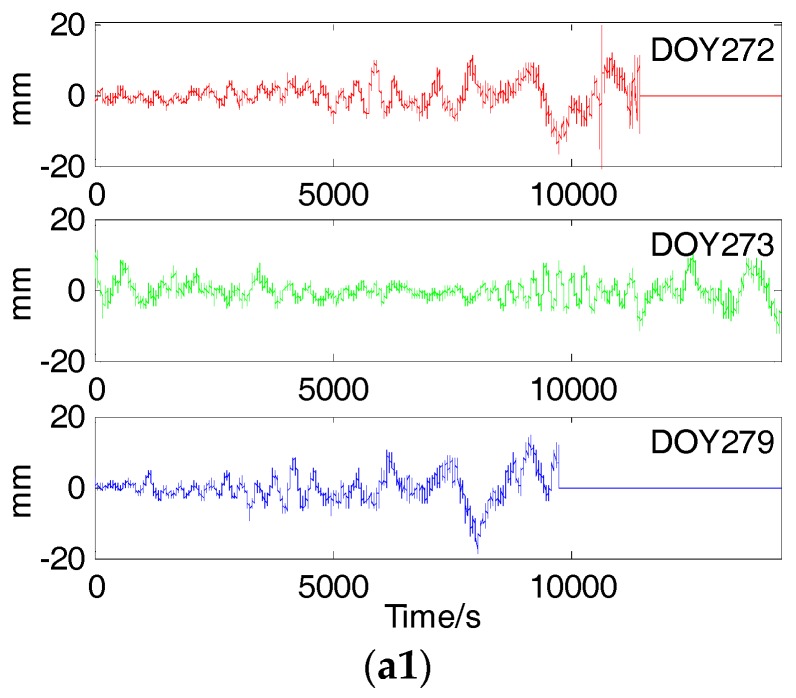

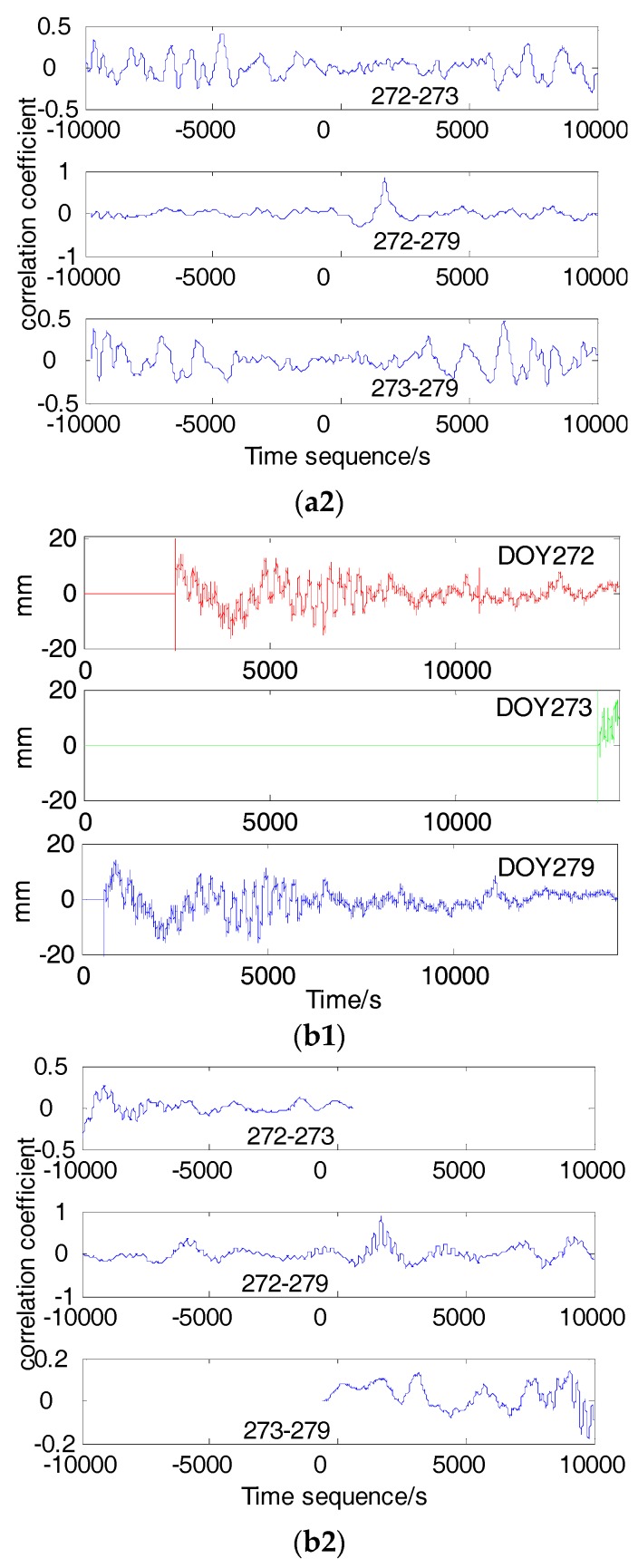
SD residuals of MEO12 (**a1**) and MEO14 (**b1**) on DOY 272, 273 and 279 and correlation curves of MEO12 (**a2**) and MEO14 (**b2**) on 272–273, 272–279 and 273–279.

According to the steps in Section 2.3, we calculated the SD residuals of different type of BDS satellites. Figure 7 shows SD residuals of GEO5 and IGSO6 on DOY 272, 273 and 274 and correlation curves of 272–273, 272–274 and 273–274. Figure 8 shows SD residuals of MEO12 and MEO14 on DOY 272, 273 and 279 and correlation curve of 272–273, 272–279 and 273–279. As we can see in the above figures, SD residuals and correlation curves of GEO5 and IGSO6 almost repeated themselves every sidereal day in the three consecutive days as satellite tracks did. This means that the periods of multipath error for BDS GEO and IGSO satellites are relevant to the corresponding orbital periods. Meanwhile, SD residuals and correlation curves of MEO12 and MEO14 are different in two consecutive days but similar between DOY 272 and 279. Similarly, periods of multipath errors for BDS MEO satellites are strong related to the corresponding orbital periods.

Table 3 and Table 4 give the orbital time shifts and residual time shifts corresponding to maximum correlation coefficients of all BDS satellites. The mean orbital time shifts of GEO and IGSO satellites are 242.6 s, 242.2 s and 484.8 s on three consecutive days, which are approximate to the mean residual time shifts where SD residuals correlation coefficients reach maximum value—239.8 s, 242.4 s and 482.3 s. What’s more, the means of correlation coefficients are up to 0.8 in spite of remaining noise in SD residuals.

### 5.2. Extracting Multipath Error Models from SD Difference Residuals

**Figure 9:**
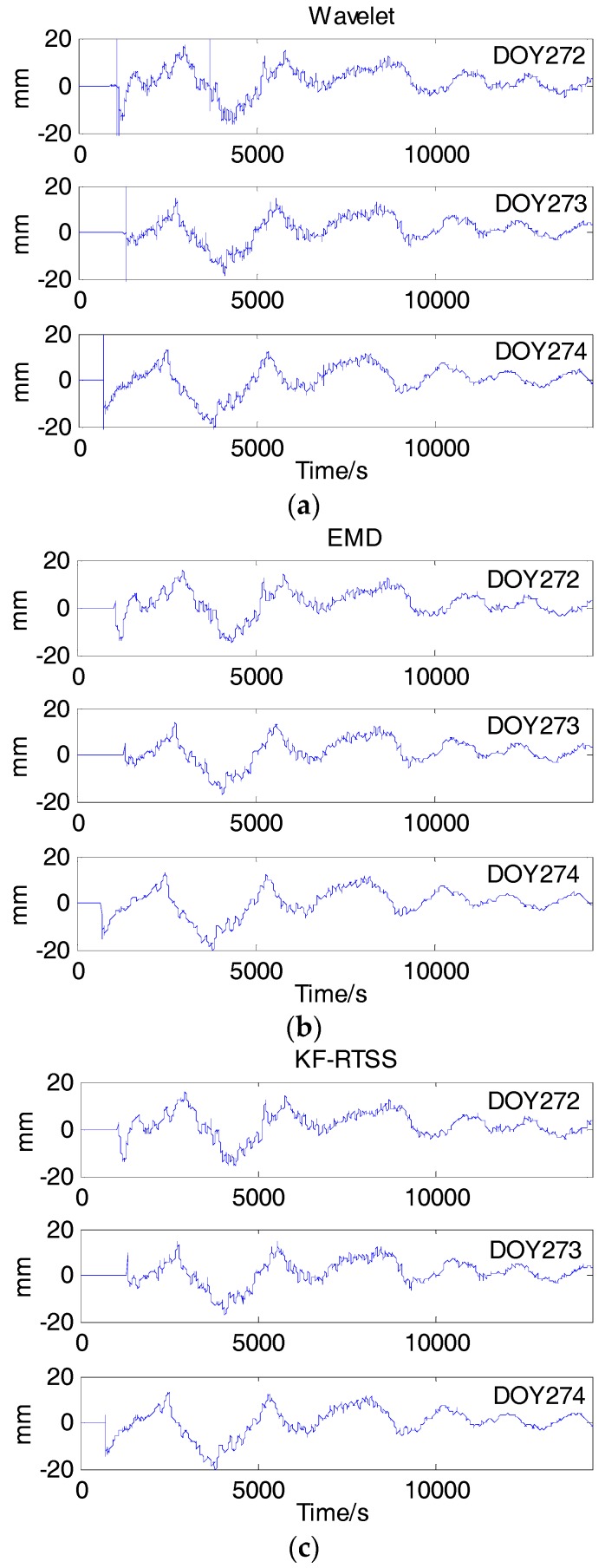
Multipath models of IGSO6 on DOY 272 (**a**), 273 (**b**), 274 (**c**) extracted from SD residuals by different filters.

The SD residuals of the last period data cannot be subtracted from SD observations of current period data because the SD residuals consist of multipath error models and white errors which are interference terms and should be filtered. Multipath models of IGSO6 for DOY 272–274 extracted from SD residuals by the three filters mentioned are shown in Figure 9. Table 5 gives the correlation coefficients of multipath models between DOY 273 and 274 for BDS PRN 1–10 satellites with three filtering methods. Compared with Table 4, we can find that the mean correlation coefficients with wavelet filter, KT-RTSS and EMD filtering are 0.9036, 0.9214 and 0.9158, respectively. These performances are all better than that of SD residuals with the value of 0.853. This indicates that three kinds of filter are all effective, therein the performance of KT-RTSS method is the best.

### 5.3. Improvement of Baseline Vector with Different Multipath Mitigation Methods

**Figure 10:**
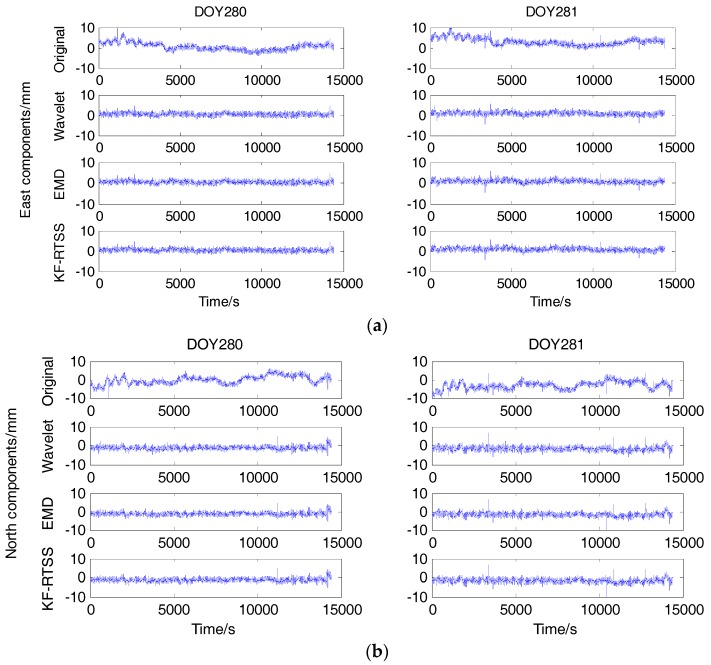

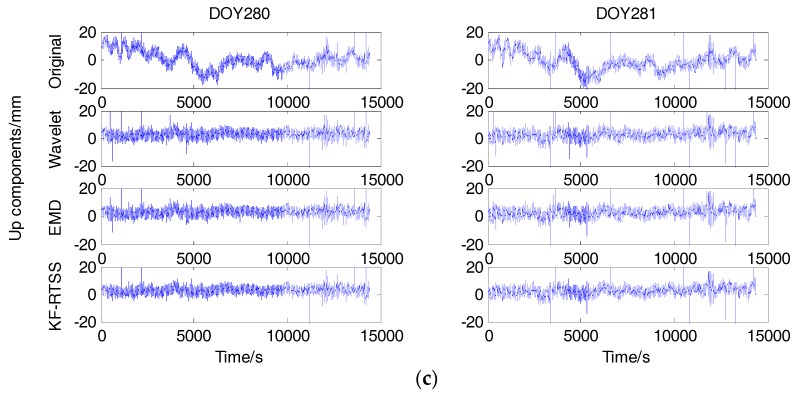
The deviation in East (**a**), North (**b**), Up (**c**) components of baseline vector corresponding to original result, Wavelet filter, EMD filter and KF-RTSS on DOY 280 and 281.

The last step of multipath mitigation is subtracting the multipath models of last period data from current period data and calculate the baseline vector. Shown in Figure 10 are the BDS baseline vectors before and after multipath mitigation with different methods. Table 6 gives the RMSE of deviations in E, N, U components and percentage of improvement respectively. The percentage of improvement with KF-RTSS method is about 62.8%, 63.6%, 62.5% and 57.3%, 53.4%, 55.9% in E, N, U components on DOY 280 and 281, which is slightly better than that of Wavelet filter and EMD filter.

## 6. Conclusions

In order to meet the requirements of deformation monitoring with BDS precise positioning techniques for large structures, it is of significance to mitigate multipath effects in carrier phase observations. In this paper, detailed analysis was provided about the characteristics of orbital periods and multipath repeat periods of BDS satellites. It was found that they are consistent. The period of orbit and multipath errors for BDS GEO and IGSO satellites is about one day and that of BDS MEO satellites is about seven days. Sidereal filters based on SD difference were applied to correct multipath errors. The KF-RTSS method was introduced to extract multipath models from single difference (SD) residuals. After subtracting multipath models, the improvement of baseline accuracy on E, N, U directions are about 62.8%, 63.6%, 62.5% on DOY 280 and 57.3%, 53.4%, 55.9% on DOY 281, respectively. Wavelet filter and EMD filter were also used in multipath mitigation. The experimental results indicate that the three filters all have obvious effect on improvement of baseline accuracy and the performance of KT-RTSS method is slightly better than that of the wavelet filter and EMD filter.

## Figures and Tables

**Table 1:** The mean, variance and range of time shift of all BDS satellites during January and February, 2017.

PRN	C01	C02	C03	C04	C05	C06	C07	C08	C09	C10	C11	C12	C14
Time shift/S	236	236	236	235	235	236	239	243	233	245	1699	1702	1703
MSE	6.0	5.3	6.0	4.9	5.3	5.3	4.8	20	3.7	3.2	2.4	2.4	3.4
Range	31	25	33	24	28	22	18	51	16	13	13	12	16

**Table 2:** Correlation coefficients and root mean square error of wavelet signal, EMD signal and KF-RTSS signal.

Filter	Original	Wavelet	EMD	KF-RTSS
correlation	1	0.9768	0.9884	0.9927
RMSE	1.236	1.294	1.269	1.278

**Table 3:** Orbital time shifts and SD residual time shifts corresponding to maximum correlation coefficients of BDS GEO and IGSO satellites.

PRN	272–273			273–274			272–274		
	Orbital Time Shift	Residual Time Shift	Maximum Correlation Coefficient	Orbital Time Shift	Residual Time Shift	Maximum Correlation Coefficient	Orbital Time Shift	Residual Time Shift	Maximum Correlation Coefficient
GEO1	237	232	0.628	237	251	0.724	474	477	0.708
GEO2	242	231	0.839	242	221	0.827	484	495	0.810
GEO3	248	244	0.894	247	243	0.916	495	494	0.884
GEO4	244	245	0.762	243	247	0.805	487	490	0.736
GEO5	239	232	0.856	239	235	0.890	478	467	0.876
IGSO6	259	253	0.818	259	263	0.881	518	506	0.786
IGSO7	220	228	0.757	219	219	0.857	439	445	0.667
IGSO8	248	247	0.884	248	252	0.903	496	491	0.888
IGSO9	255	257	0.854	254	259	0.857	509	495	0.807
IGSO10	234	229	0.822	234	234	0.867	468	463	0.832
Mean	242.6	239.8	0.811	242.2	242.4	0.853	484.8	482.3	0.799

**Table 4:** Orbital time shift and Residual time shift corresponding to maximum correlation coefficients of BDS MEO satellites.

PRN	272–273			272–279			273–279		
	Orbital Time Shift	Residual Time Shift	Maximum Correlation Coefficient	Orbital Time Shift	Residual Time Shift	Maximum Correlation Coefficient	Orbital Time Shift	Residual Time Shift	Maximum Correlation Coefficient
MEO12	-	−4649	0.413	1704	1705	0.834	-	6389	0.467
MEO14	-	−9923	0.267	1702	1699	0.888	-	9102	0.142

**Table 5:** Correlation coefficients of multipath models between DOY 273 and 274 for all BDS satellites after three filters.

PRN	Wavelet	KT-RTSS	EMD
	Maximum Correlation Coefficient	Maximum Correlation Coefficient	Maximum Correlation Coefficient
GEO1	0.8526	0.8888	0.8732
GEO2	0.9207	0.9290	0.9298
GEO3	0.9631	0.9723	0.9686
GEO4	0.8564	0.8780	0.8741
GEO5	0.9631	0.9587	0.9611
IGSO6	0.9003	0.9286	0.9261
IGSO7	0.8943	0.9079	0.9011
IGSO8	0.9418	0.9482	0.9472
IGSO9	0.8796	0.9081	0.9021
IGSO10	0.8642	0.8945	0.8749
Mean	0.9036	0.9214	0.9158

**Table 6:** The deviation in E, N, U components of baseline vector corresponding to original result, Wavelet filter, EMD filter and KF-RTSS and improvement in E, N, U components of baseline vector corresponding to Wavelet filter, EMD filter and KF-RTSS than original result on DOY 280 and 281.

DOY		280				281			
Filter		Original	Wavelet	EMD	KF-RTSS	Original	Wavelet	EMD	KF-RTSS
RMSE/mm	E	1.817	0.6908	0.6856	0.6754	1.733	0.7538	0.7531	0.7406
	N	2.165	0.8085	0.8028	0.7876	2.023	0.9548	0.956	0.9429
	U	6.112	2.386	2.387	2.294	6.331	2.86	2.876	2.795
Percentage of improvement	E	-	61.9%	62.3%	62.8%	-	56.5%	56.6%	57.3%
	N	-	62.6%	62.9%	63.6%	-	52.8%	52.7%	53.4%
	U	-	60.9%	60.9%	62.5%	-	54.8%	54.5%	55.9%
